# Diaschisis revisited: quantitative evaluation of thalamic hypoperfusion in anterior circulation stroke

**DOI:** 10.1016/j.nicl.2020.102329

**Published:** 2020-06-26

**Authors:** Paul Reidler, Franziska Mueller, Lena Stueckelschweiger, Katharina Feil, Lars Kellert, Matthias P. Fabritius, Thomas Liebig, Steffen Tiedt, Daniel Puhr-Westerheide, Wolfgang G. Kunz

**Affiliations:** a Department of Radiology, University Hospital, LMU Munich, Germany; b Department of Neurology, University Hospital, LMU Munich, Germany; c Department of Neuroradiology, University Hospital, LMU Munich, Germany; d Institute for Stroke and Dementia Research, LMU Munich, Germany

**Keywords:** ASPECTS, Alberta Stroke Program Early CT Score, CTP, CT perfusion, ITD, ipsilateral thalamic diaschisis, LVO, large vessel occlusion, MCA, medial cerebral artery, mTICI, modified treatment in cerebral ischemia, PCA, posterior cerebral artery, Stroke, Cerebrovascular circulation, Computed tomography, Cerebral ischemia, Thalamus

## Abstract

**Purpose:**

Ipsilateral thalamic diaschisis (ITD) refers to the phenomenon of thalamic hypoperfusion or hypometabolism due to a distant cerebral injury. To further investigate the characteristics and spectrum of ITD, we analyzed quantitative measurements of thalamic hypoperfusion in acute anterior circulation stroke.

**Methods:**

We selected consecutive patients with large-vessel occlusion (LVO) anterior circulation stroke and available CT perfusion (CTP) examination on admission who underwent endovascular thrombectomy. Thalamic perfusion parameters on CTP were tested between ipsi- and contralesional thalamus and ischemic territory. Values were compared with thresholds from CTP analysis software. Associations of thalamic perfusion parameters with acute imaging and clinical data were determined in uni- and multivariate logistic regression analyses.

**Results:**

Ninety-nine patients were included. All perfusion parameters indicated significant non-ischemic hypoperfusion of the thalamus, not reaching the levels of ischemia in the middle cerebral artery territory due to LVO (all p < 0.002). Multiple perfusion parameters exhibited significant association with ischemic lesion extent (relative cerebral blood flow [CBF]: β =  − 0.23, p = 0.022; Δtime to drain: β = 0.33, p < 0.001; ΔTmax: β =  − 0.36, p < 0.001) and involvement of the Lentiform Nucleus (Δmean transit time: β = 0.64, p = 0.04; Δtime to drain: β = 0.81, p = 0.01; ΔTmax: β =  − 0.82, p = 0.01). Symptom severity on admission exhibited minor significant association with reduction of thalamic CBF in uncorrected analysis (Odds ratio: 0.05, p = 0.049), but short- and long-term outcomes were unaffected by perfusion status. ITD reached guideline-based software-threshold levels in only one patient.

**Conclusions:**

ITD in acute stroke is a non-binary phenomenon affected by lesion extent and involvement of the lentiform nucleus. We found uncorrected association of ITD with early clinical presentation, but no association with short- or long-term outcome was evident. Relevant misclassification of ITD by guideline-based CTP software was not indicated, which needs further dedicated testing.

## Highlights

• CT perfusion reveals thalamic hypoperfusion in acute anterior circulation stroke.
• This indirect phenomenon is referred to as ipsilateral thalamic diaschisis (ITD).
• Quantitative analysis indicates that ITD is a non-binary phenomenon.
• ITD is associated with lesion extent and involvement of the lentiform nucleus.
• Stroke outcome was not associated with ITD parameters.

## 1 Introduction

Ipsilateral thalamic diaschisis (ITD) refers to the phenomenon of thalamic hypoperfusion, hypometabolism, hypofunction and loss of volume ipsilateral to a distant cerebral injury. (Carrera and Tononi, 2014) ITD is especially known to occur due to supratentorial cerebrovascular injury (Fiorelli et al., 1991, Ogawa et al., 1997, Sakashita et al., 1993, De Reuck et al., 1995, Craig et al., 2019, Hendrik, 2020). The implicated mechanisms of ITD include defective transmission along corticothalamic or thalamocortical pathways in the acute phase and transneuronal degeneration in the chronic phase (Duering and Schmidt, 2017).

In the setting of anterior circulation stroke, thalamic hypoperfusion is not expected as the thalamus is supplied by the vertebrobasilar system, especially by perforators of the posterior cerebral artery (PCA) (Schmahmann, 2003). Yet, ipsilesional thalamic hypoperfusion and hypometabolism can be found in the acute (Reidler et al., 2018), subacute (Fiorelli et al., 1991, Sakashita et al., 1993, Hendrik, 2020) and chronic (De Reuck et al., 1995) phase of stroke. Numerous studies established the use of positron emission tomography (PET) or magnet resonance imaging (MRI) to examine diaschisis phenomena, predominantly in later phases (Baron et al., 1981, Sebok et al., 2018). Alongside these modalities, 4-dimensional, dynamic CT perfusion (CTP), as used in critical stroke care, advanced the visualization of diaschisis phenomena to the acute stroke phase by sampling contrast enhancement dynamics of brain tissue (Sommer et al., 2016, Kunz et al., 2017, Reidler et al., 2018).

At the time, studies on ITD present discord. Acute stroke patients with positive ITD status on CTP displayed larger total ischemic and ischemic core volumes as well as a more frequent involvement of subcortical structures (Reidler et al., 2018). Acute ITD occurred less frequently compared to later-performed PET or MRI studies (Fiorelli et al., 1991, Sakashita et al., 1993, De Reuck et al., 1995, Hendrik, 2020). Further, the majority of studies on acute or chronic stroke patients found no impact of ITD (De Reuck et al., 1995) on clinical outcome, while other studies suggest inferior outcome in ITD positive patients (Hendrik, 2020, Binkofski et al., 1996). ITD on CTP can moreover cause uncertainty in acute clinical decision making, hence software-based CTP threshold values, which gained status as main selection criterion for late time-window thrombectomy, might be biased by thalamic hypoperfusion (Reidler et al., 2018, Nogueira et al., 2018, Albers et al., 2018, Powers et al., 2019).

While some studies applied quantitative thresholds for binary ITD classification (Hendrik, 2020, De Reuck et al., 1995), the degree of thalamic hypoperfusion/hypometabolism as a quantitative, scaled biomarker, and its impact on imaging and clinical features remains uncertain. Data on thalamic and crossed cerebellar diaschisis or chronic iron deposition in the thalamus after supratentorial stroke indicate the presence of a spectrum of severity in diaschisis phenomena and not necessarily discrete states (Sebok et al., 2018, Hendrik, 2020, Sommer et al., 2016, Kuchcinski et al., 2017).

To test this hypothesis, we applied quantitative analysis of thalamic perfusion on CTP data in patients with acute anterior circulation stroke and analyzed its association with acute imaging parameters as well as clinical outcome measures after thrombectomy. Further, we compared our results with current thresholds used in CTP analysis software.

## 2 Materials and methods

### 2.1 Study design and population

This study was approved by the institutional review board of the LMU Munich according to the Declaration of Helsinki of 2013. Subjects were selected from two prospectively acquired cohorts using the same selection criteria: The first cohort consisted of 1,644 patients who underwent multiparametric CT including CT perfusion (CTP) for suspected stroke between 2009 and 2014. The second cohort consisted of 274 consecutive stroke patients between 2015 and 2017 treated with endovascular thrombectomy (EVT).

For our retrospective analysis we included patients with:

(1) internal carotid artery, M1 or M2 segment artery occlusion,
(2) complete noncontrast CT, CT angiography, and CTP imaging data including raw 4D CTP dataset
(3) treated with endovascular thrombectomy

We excluded patients with:

(1) prior ischemia,
(2) pathology of the posterior circulation
(3) intracranial mass, that might present an epileptogenic focus.

Applying the criteria resulted in the inclusion of 20 patients from the first cohort and 79 patients from the second cohort to a total of 99 patients. Patients from the first cohort were reported before in a study on ITD using visual classification (Reidler et al., 2018). All patients were reported before in a study on automated attenuation analysis on non-contrast CT data (Reidler et al., 2019). A detailed flow-chart of patient selection is provided in Figure I of the Supplementary Material.

### 2.2 CT acquisition

Emergency imaging protocol included non-contrast CT, CT angiography (CTA) from aortic arch to the vertex, and CT perfusion (CTP). Scans were performed on SOMATOM Definition AS+, SOMATOM Definition Flash and SOMATOM Definition Edge scanners (Siemens Healthcare, Forchheim, Germany). CTP was obtained with 100-mm scan coverage in the z-axis, continuously over 48 s (32 cycles, one sweep every 1.5 s). 80 kV voltage and 200 mAs current was applied. 35 mL of iodinated contrast agent (400 mg/mL) was administered intravenously at a flow rate of 5 mL/s, followed by a saline flush of 40 mL at 5 mL/s. Follow-up imaging consisted of magnetic resonance imaging or noncontrast CT.

### 2.3 Image analysis

Regional distribution of acute ischemia was determined according to the Alberta Stroke Program Early CT Score (ASPECTS) on CTP cerebral blood flow (CBF) maps as described before by two blinded readers (Reidler et al., 2019). A region was rated positive if ≥ 20% of the regional volume was affected, in accordance with other studies (d'Esterre et al., 2017). Further, we analyzed CTA data for the presence of a persisting fetal posterior cerebral artery (PCA) as a possible variant of thalamic blood supply (Dimmick and Faulder, 2009). Initial total ischemic volume, ischemic core volume and final infarction volume were manually segmented on CBF maps, cerebral blood volume (CBV) maps, and follow-up noncontrast CT or MRI, respectively, using commercial software (OsiriX v.8.0.2, Pixmeo 2017).

### 2.4 CT perfusion analysis

**Fig. 1:**
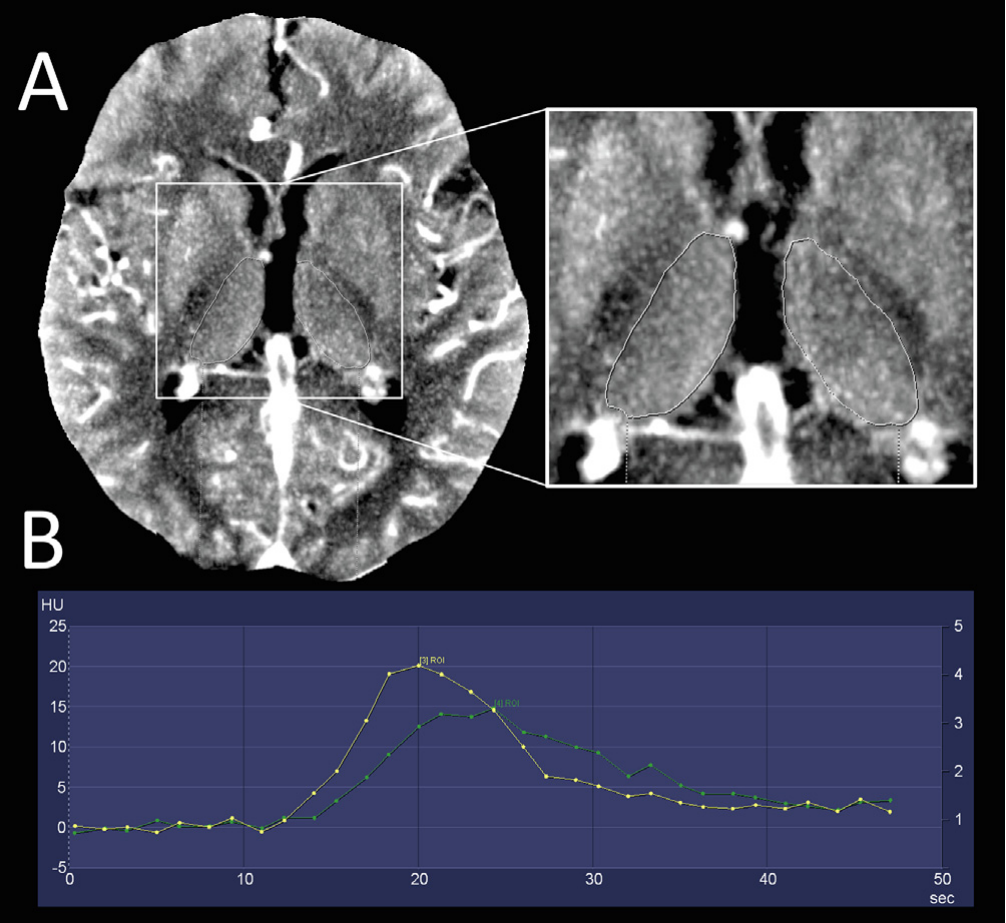
Measurements of thalamic perfusion parameters on temporal MIP CTP images. A) Example of manual segmentation of both thalami in a patient with left M1 occlusion. Right: Enlarged section with segmented thalamic outline (white) on both sides. B) Derived tissue attenuation curves of ipsilesional ROI (green) and contralesional ROI (yellow). Abbreviations: MIP, maximum intensity projection; CTP, CT perfusion; ROI, region of interest; HU, Hounsfield units. (For interpretation of the references to colour in this figure legend, the reader is referred to the web version of this article.)

CTP data were processed and analyzed using the CT vendors dedicated software (syngo Neuro Perfusion CT, Siemens Healthineers, Forchheim, Germany) as described in detail before (Thierfelder et al., 2013). Individual manual segmentation of the thalami was carried out on representative axial slices on temporal maximum intensity projection (MIP) reconstructions of raw CTP data (see Fig. 1). Clipping of adjacent arterial and venous vasculature was avoided to ensure unbiased measurements of tissue perfusion. Correct segmentation was confirmed by expert readers (P.R., W.G.K.). Cerebral blood flow (CBF) in ml/100 g/min, cerebral blood volume (CBV) in ml/100 g, as well as time to drain (TTD), mean transit time (MTT) and TMAX in seconds were derived from the perfusion curves of both thalami using the softwares automated deconvolution algorithm (Konstas et al., 2009a, Konstas et al., 2009b, Allmendinger et al., 2012, Abels et al., 2010). To determine perfusion reduction, relative CBF (rCBF), CBV (rCBV), MTT (rMTT), TTD (rTTD) and TMAX (rTMAX) were defined as ratio of ipsilesional vs. contralesional measurements. ΔTTD, ΔMTT and ΔTMAX were defined as time difference between ipsilesional and contralesional measurements in seconds. The same procedures were applied to the ischemic territory and corresponding area of the contralesional hemisphere on representative axial slices on CBF maps with the largest extent of ischemia. Results of thalamic hypoperfusion were compared to threshold values of the current CTP analysis software packages RAPID (iSchemaView, USA), Syngo Via Neuro Perfusion (Siemens Healthineers, Forchheim, Germany) and Brain CT Perfusion Package (Philips Healthcare, Best, The Netherlands) (Austein et al., 2016).

### 2.5 Clinical outcome data

Clinical outcome measures were determined by means of the National Institutes of Health Stroke Scale (NIHSS) on admission as well as on the modified Rankin Scale (mRS) at discharge and 90 days after stroke. Patients without sufficient records, premorbid mRS > 1 or death due to other cause within 90 days were excluded from the clinical outcome analysis. This resulted in 83 valid cases for short-term (discharge mRS) and 70 valid cases for long-term (90-day mRS) outcome assessment.

### 2.6 Statistical analysis

Statistical analysis was performed using SPSS Statistics 24 (IBM, Armonk/NY, USA). Normality was determined by the Shapiro-Wilk Test. Mann-Whitney U Test was performed to determine significant differences of perfusion measurements due to non-normal distribution. Univariate and multivariate linear regression analysis was performed to find associations of thalamic perfusion measurements with imaging parameters and lesion location. Analysis for clinical outcome as dependent variables used ordinal logistic regression. Multicollinearity of independent variables was tested using the variance inflation factor to avoid overfitting of the regression models. Statistical significance was defined for p values lower than 0.05. Bonferroni correction was used to account for multiple comparisons.

## 3 Results

### 3.1 Patient characteristics

Ninety-nine patients were included. Median age was 75 years (interquartile range [IQR]: 63 – 81), 50 female patients were included (50.5%). Most frequent site of large vessel occlusion was the M1 segment (87.9%) followed by the M2 segment (49.5%) of the middle cerebral artery (MCA). On admission, patients presented with a median noncontrast CT ASPECTS of 8 (IQR: 7 – 10) and NIHSS of 14 (IQR: 9 – 17). According to our inclusion criteria all patients were treated with EVT, 67 patients (67.7%) were additionally treated with intravenous thrombolysis. Median final infarction volume in the MCA territory was 21 mL (IQR: 7– 73). No patients displayed damage to the thalamus on follow-up. Detailed patient characteristics are presented in Table 1.

**Table 1:** Patient characteristics.

	**N = 99**	
**Patient Data**		
Age	75	(63–81)
Female sex	50	(50.5%)
Time from symptom onset	89	(67–135)
NIHSS on admission	14	(9–17)
**Treatment**		
IV thrombolysis	67	(67.7%)
Endovascular thrombectomy	99	(100%)
**Imaging**		
Noncontrast CT ASPECTS	8	(7–10)
Occluded vessels		
ICA	28	(28.3%)
Carotid T	22	(22.2%)
M1 segment of MCA	87	(87.9%)
M2 segment of MCA	49	(49.5%)
Total ischemic volume	143	(108–199)
Infarction core volume	17	(10–47)
CTP Mismatch %	85	(71–93)
Final infarction volume	21	(7–73)
Fetal PCA	9	(9.1%)
**Complications**		
Hemorrhagic infarction	16	16.2%
Parenchymal hematoma	8	8.1%
Space-occupying edema	8	(8.1%)
**Clinical Analysis**		
Premorbid mRS	0	(0–1)
Discharge mRS	4	(3–5)
90-day mRS	3	(1–6)

Values presented are count (percentage) for categorical and median (interquartile range) for ordinal or continuous variables. Time values are presented in minutes, volume values as mL. Abbreviations: NIHSS, national Institute of Health Stroke Scale; IV, intravenous; ASPECTS, Alberta Stroke Program Early CT Score; ICA, internal carotid artery; MCA, middle cerebral artery; CTP; CT perfusion; PCA, posterior cerebral artery; mRS, modified Rankin Scale.

### 3.2 Perfusion analysis of thalamus and ischemic territory

All parameters indicate significant hypoperfusion of the ipsilesional thalamus compared to the contralesional side with reduction of CBF and CBV (p < 0.001) and elevation of MTT (p = 0.001), TTD and TMAX (each p < 0.001). As expected, same observations are made between the ischemic territory and the corresponding area of the contralesional hemisphere (each p < 0.001, except CBV (p = 0.001). Most relative (ipsi- by / minus contralesional hemisphere) measurements of thalamic hypoperfusion did not reach levels of true ischemia (each p < 0.001), only rCBV in the thalamus presented a slightly marked decrease (0.84 [IQR: 0.75 – 0.92] vs. 0.92 [IQR: 0.80–1.04], p = 0.002). Statistical significance is maintained for all parameters considering correction for multiple comparisons with Bonferronís method. Detailed results of perfusion analysis are presented in Table 2. Subgroup analysis in patients without fetal PCA provides similar results and is provided in Supplementary Material Table I. In an exploratory analysis we have selected consecutive stroke patients who underwent multiparametric CT including CT perfusion but did not demonstrate ischemic changes on acute or follow-up imaging. We measured thalamic perfusion in this cohort and calculated relative parameters. This analysis shows statistically significant differences between our study cohort and stroke negative patients. Using these measurements as cut-off values would result in classification of up to 63% of patients as ITD positive. Detailed results are displayed in Supplementary Material Table II and III.

**Table 2:** Perfusion parameters of thalamus and ischemic territory.

Absolute Measurements			
N = 99	Ipsilesional Thalamus	Contralesional Thalamus	p value
CBF [mL/100 g/min]	55.5 (48.5–63.7)	71.2 (62.1–78.4)	<0.001*
CBV [mL/100 g]	3.35 (3.06–3.68)	4.01 (3.61–4.40)	<0.001*
MTT [s]	3.89 (3.46–4.61)	3.45 (3.14–4.05)	0.001*
TTD [s]	3.40 (2.21–4.57)	2.37 (1.52–3.46)	<0.001*
TMAX [s]	1.49 (0.53–2.35)	0.70 (0.14–1.52)	<0.001*
**N = 99**	**Ischemic Territory**	**Contralesional Territory**	**p value**
CBF [mL/100 g/min]	27.8 (21.1–36.4)	59.9 (54.2–67.7)	<0.001*
CBV [mL/100 g]	3.27 (2.79–3.72)	3.52 (3.29–4.60)	0.001*
MTT [s]	8.66 (7.31–10.5)	3.96 (3.41–4.41)	<0.001*
TTD [s]	11.99 (10.8–14.1)	3.28 (2.23–2.11)	<0.001*
TMAX [s]	7.82 (6.50–9.53)	1.36 (0.63–3.75)	<0.001*
**Relative Measurements**			
**N = 99**	**Thalamus**	**Ischemic/Contralesional Territory**	**p value**
			<0.001
rCBF	0.78 (0.70–0.89)	0.49 (0.36–0.60)	<0.001*
rCBV	0.84 (0.75–0.92)	0.92 (0.80–1.04)	0.002*
rMTT	1.06 (1.00–1.17)	2.08 (1.71–2.59)	<0.001*
rTTD	1.30 (1.16–1.56)	3.51 (2.73–5.30)	<0.001*
rTMAX	1.70 (1.38–2.70)	5.51 (3.88–10.4)	<0.001*
ΔMTT [s]	0.21 (0.01–0.55)	4.54 (3.18–6.05)	<0.001*
ΔTTD [s]	0.72 (0.34–1.25)	8.87 (7.06–10.7)	<0.001*
ΔTMAX [s]	0.53 (0.24–0.92)	6.31 (5.34–7.63)	<0.001*

Values presented are median (interquartile range). Nonparametric tests were performed using the Mann-Whitney *U* test Abbreviations: CBF, cerebral blood flow; CBV, cerebral blood volume; MTT, mean transit time; TTD, time to drain; rCBF/rCBV/rMTT/rTTD/rTMAX, relative ratio between ipsi- and contralesional measurements; ΔTTD / ΔMTT / ΔTMAX, absolute difference between ipsi- minus contralateral measurements. Bold numbers indicate p < 0.05. * statistically significant after Bonferroni correction for 18 comparisons.

### 3.3 Comparison of perfusion characteristics with CTP analysis software thresholds

In our cohort, we did not find patients with rCBV < 30% and only one patient with TMAX slightly > 6 s (TMAX = 6.2 s) as used for ischemic core and hypoperfusion classification in RAPID (iSchemaView, USA). Absolute thresholds of CBF < 35.1 mL/100 g/min for hypoperfusion or CBV < 1.2 mL/100 mg for ischemic core, as used in our CT vendoŕs software (syngo Neuro Perfusion CT, Siemens Healthineers, Forchheim, Germany) were only reached in one patient. Thresholds of relative MTT > 145% for ischemic hypoperfusion as used in Brain CT Perfusion Package (Philips Healthcare, Best, The Netherlands) were reached in 5 / 99 patients, the softwarés thresholds for ischemic core (relative MTT > 145% and CBV < 2.0 mL/100 g) were reached in 0 patients (Austein et al., 2016). Detailed results are displayed in Table IV of the Supplementary Material.

### 3.4 Association of thalamic hypoperfusion with acute imaging parameters

Reduction of thalamic rCBF presented an uncorrected significant association with larger total ischemic volume (β = -0.23, p = 0.022), infarction core volume (β = -0.22, p = 0.031) and lower noncontrast ASPECTS (β = -0.21, p = 0.04) for univariate linear regression analysis.

Reduction of thalamic rCBV presented an uncorrected significant association with larger infarction core volume (β = -0.24, p = 0.02), lower CTP mismatch percentage (β = 0.21, p = 0.04) and Bonferroni corrected significant association with lower noncontrast ASPECTS (β = 0.22, p = 0.001) for univariate linear regression.

Increase in thalamic ΔTTD and ΔTMAX presented a corrected significant association with larger total ischemic volume (β = 0.33, p = 0.001 and β = 0.36, p < 0.001) and additionally uncorrected significant association for ΔTMAX with larger ischemic core volume (β = 0.36, p = 0.048).

Further, uncorrected significant associations were shown in multivariate linear regression analysis between thalamic rCBV (β = 0.27, p = 0.02) and noncontrast CT ASPECTS and between total ischemic volume and thalamic ΔMTT(β = 0.25, p = 0.05), with corrected significant results for association of total ischemic volume and ΔTTD (β) = 0.34, p = 0.004) and ΔTMAX (β = 0.36, p = 0.002). Presence of fetal PCA did not present any significant association with thalamic perfusion parameters (all with p > 0.3). Detailed results are presented in Table 3. Scatter plots are provided in Figure II and additional results of the multivariate linear regression analysis are provided in Table V of the Supplementary Material. Subgroup analysis in patients without fetal PCA provides similar results and is provided in Supplementary Material Table VI.

**Table 3:** Association of thalamic perfusion with acute imaging parameters.

N = 99	Thalamic rCBF		Thalamic rCBV		Thalamic ΔMTT		Thalamic ΔTTD		Thalamic ΔTMAX	
**Independent Variables**	**β**	**p value**	**β**	**p value**	**β**	**p value**	**β**	**p value**	**β**	**p value**
Total ischemic volume	−0.23	**0.022**	−0.18	0.08	0.19	0.07*	0.33	0.001*^†‡^	0.36	<0.001*^†‡^
Ischemic core volume	−0.22	**0.031**	−0.24	**0.02**	0.03	0.75	0.19	0.06	0.20	**0.048**
Mismatch %	0.15	0.12	0.21	**0.04**	0.05	0.66	−0.06	0.56	0.07	0.50
Noncontrast CT ASPECTS	0.21	**0.04**	0.22	0.001*^†‡^	0.10	0.32	−0.03	0.77	−0.09	0.28
Fetal PCA	−0.40	0.74	−0.03	0.70	−0.04	0.70	−0.04	0.72	−0.05	0.62

Univariate and multivariate linear regression analyses were performed for the indicated acute imaging parameters. Presented are the results of the univariate analysis. *p < 0.05 in multivariate linear regression analysis additionally incorporating total ischemic / ischemic core volume, Noncontrast CT ASPECTS and fetal PCA. Abbreviations: CBF, cerebral blood flow; CBV, cerebral blood volume; MTT, mean transit time; TTD, time to drain; rCBF / rCBV, relative CBF / CBV as ratio between ipsi- and contralesional measurements; ΔTTD / ΔMTT / ΔTMAX, absolute difference between ipsi- minus contralateral measurements, ASPECTS, Alberta Stroke Program Early CT Score, PCA, posterior cerebral artery. Bold numbers indicate p < 0.05. † statistically significant after Bonferroni correction across 5 regression models. ‡ statistically significant after Bonferroni correction for 25 parameters.

### 3.5 Association of thalamic hypoperfusion with stroke topography

**Fig. 2:**
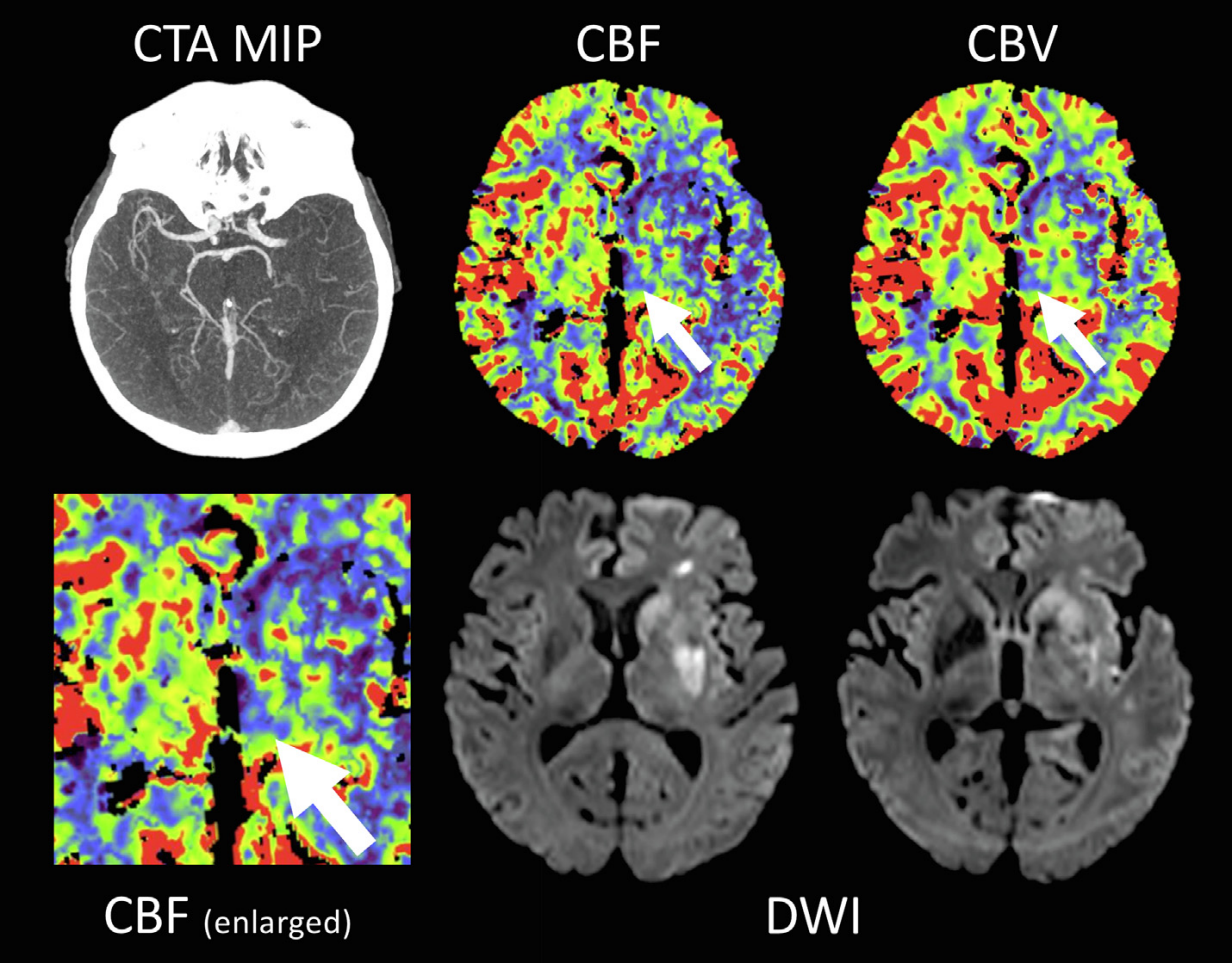
Example of a 74 year-old, male patient with left sided M1-occlusion (same as in Fig. 1). CTA presents proximal M1-occlusion on the left side with consecutive perfusion deficit in the left MCA territory on CTP. Additionally, marked thalamic hypoperfusion adjacent to the third ventricle is visible on the ipsilesional side (white arrows). Follow-up DWI after 6 days presents ischemic damage to the basal ganglia, but not to the thalamus. Also note absence of fetal PCA on CTA. Abbreviations: CTA, CT angiography; MIP, maximum intensity projection; CBF, cerebral blood flow; CBV, cerebral blood volume; DWI, diffusion weighted imaging; CTP, CT perfusion; PCA, posterior cerebral artery.

Multivariate linear regression analysis including binary involvement of all ASPECTS regions revealed a positive association between Ischemia of the Lentiform Nucleus and larger thalamic ΔMTT (β = 0.65, p = 0.04), ΔTTD (β = 0.81, p = 0.01), and ΔTMAX (β = 0.82, p = 0.01). While association with ΔMTT could not retain significance after correction for multiple comparisons, association with ΔTTD and ΔTMAX remained significant after correction across regression models but not across all parameters. None of the other ASPECTS regions displayed any significant influence on thalamic perfusion parameters. Thalamic rCBF and rCBV did not present any significant dependence on lesion location. Detailed results are presented in Table 4. A case example with involvement of the basal ganglia is displayed in Fig. 2. Topography of acute ischemia and final infarction are displayed in Supplementary Material Table VII.

**Table 4:** Association of thalamic perfusion with acute stroke topography.

**N = 99**	**ThalamicrCBF**		**ThalamicrCBV**		**Thalamic** Δ**MTT**		**Thalamic** Δ**TTD**		**Thalamic** Δ**TMAX**	
							
**IndependentVariables**	**β**	**p value**	**β**	**p value**	**β**	**p value**	**β**	**p value**	**β**	**p value**
Caudate Nucleus	0.25	0.19	0.08	0.66	−0.23	0.23	−0.22	0.24	−0.20	0.29
Internal Capsule	−0.11	0.66	−0.11	0.68	−0.38	0.16	−0.44	0.09	−0.45	0.09
Insula	−0.03	0.81	−0.20	0.17	−0.20	0.17	−0.12	0.42	−0.08	0.58
Lentiform Nucleus	−0.26	0.38	−0.08	0.79	0.64	**0.04**	0.81	**0.01^†^**	0.82	**0.01^†^**
M1 Cortex	0.00	0.98	0.09	0.60	0.17	0.33	0.18	0.29	0.17	0.32
M2 Cortex	−0.02	0.89	−0.02	0.88	−0.03	0.81	−0.04	0.75	−0.04	0.72
M3 Cortex	0.03	0.86	0.02	0.92	0.01	0.95	0.07	0.68	0.08	0.67
M4 Cortex	−0.22	0.16	−0.18	0.27	−0.01	0.97	−0.02	0.92	−0.04	0.82
M5 Cortex	0.04	0.72	−0.02	0.84	−0.04	0.73	0.05	0.68	0.09	0.41
M6 Cortex	−0.06	0.73	0.02	0.91	0.17	0.37	0.06	0.75	0.02	0.91
							
Multivariate linear regression analyses were performed for regional presence of ischemia on acute CTP imaging for the indicated regions of the ASPECTS score. Abbreviations: CBF, cerebral blood flow; CBV, cerebral blood volume; MTT, mean transit time; TTD, time to drain; rCBF / rCBV, relative CBF / CBV as ratio between ipsi- and contralesional measurements; ΔTTD / ΔMTT / ΔTMAX, absolute difference between ipsi- minus contralateral measurements; ASPECTS, Alberta Stroke Program Early CT Score; Bold numbers indicate p < 0.05. † statistically significant after Bonferroni correction across 5 regression models. ‡ statistically significant after Bonferroni correction for 50 parameters.

### 3.6 Association of thalamic perfusion with clinical parameters

To test for associations with clinical parameters, relative thalamic perfusion measures were separately included in different ordinal regression models. The model for admission NIHSS was corrected for age, sex, noncontrast CT ASPECTS as well as total ischemic and ischemic core volume. For discharge mRS and 90-day mRS we included age, sex, CBF deficit volume, final infarction volume, modified treatment in cerebral ischemia score (mTICI) as measurement of recanalization after EVT and intravenous therapy status as covariates.

For admission NIHSS we found a marginally significant independent association between lower thalamic rCBF and larger NIHSS (OR: 0.05, p = 0.049) in uncorrected analysis including all 99 patients. Statistical significance was not maintained after correction for multiple comparisons. None of the thalamic perfusion measurements on acute CTP did present significant influence on mRS at discharge in 83 analyzed patients or after 90 days in 70 analyzed patients. Sex was not significantly associated with stroke outcome in any of the analysis. Detailed results are displayed in Table 5. Results for the applied regression models without thalamic perfusion parameters are presented in Table VIII of the Supplementary Material. Here, as expected, extent of acute ischemia, final infarction, age and mTICI presented significant influence on the different parameters (p=<0.001 – 0.03).

**Table 5:** Association of thalamic perfusion with clinical parameters.

	**AdmissionNIHSS (N = 99)**		**DischargemRS (n = 83)**		**90-DaymRS (n = 70)**	
**IndependentVariables**	**OR(95%-CI)**	**p value**	**OR(95%-CI)**	**p value**	**OR(95%-CI)**	**p value**
rCBF	0.05 (0.01 – 0.98)	**0.049**	0.09 (0.01 – 3.51)	0.20	0.09 (0.01 – 4.66)	0.24
rCBV	0.06 (0.01 – 1.38)	0.08	0.31 (0.08 – 1.1)	0.52	0.42 (0.01 – 20.2)	0.66
ΔMTT [s]	1.14 (0.71 – 1.84)	0.60	2.11 (0.69 – 2.27)	0.46	1.55 (0.82 – 2.94)	0.18
ΔTTD [s]	1.13 (0.82 – 1.55)	0.45	1.15 (0.78– 1.59)	0.55	1.15 (0.79 – 1.67)	0.48
ΔTMAX [s]	1.17 (0.77 – 1.77)	0.48	1.64 (0.68 – 1.73)	0.74	1.30 (0.71 – 1.90)	0.56

A multivariable, ordinal logistic regression analysis was performed for the indicated parameters. Further variables for analysis of Admission NIHSS included Age, Sex, CBF / CBV deficit volume, Noncontrast CT ASPECTS. Further variables for Discharge and 90-day mRS included Age, Sex, Total ischemic volume, final infarction volume, mTICI after Thrombectomy, intravenous therapy. Abbreviations: NIHSS, National Institutes of Health Stroke Scale; mRS, modified Rankin Scale; CBF, cerebral blood flow; CBV, cerebral blood volume; MTT, mean transit time; TTD, time to drain; rCBF / rCBV, relative CBF / CBV as ratio between ipsi- and contralesional measurements; ΔTTD / ΔMTT / ΔTMAX, absolute difference between ipsi- minus contralateral measurements; OR, odds ratio, mTICI, modified Treatment in Cerebral Ischemia score. Bold numbers indicate p < 0.05. † statistically significant after Bonferroni correction across 3 regression models. ‡ statistically significant after Bonferroni correction for 15 parameters.

To further delineate clinical impact of ITD from therapy effects, we have performed a regression analysis for clinical outcome parameters in subgroups with successful (mTICI 2b/3) or unsuccessful reperfusion (mTICI 0-2a). This analysis in smaller subgroups did not present significant effects of ITD on clinical symptoms. Results are displayed in Supplementary Material Table IX and X.

## 4 Discussion

Our study introduces the first comprehensive, quantitative data on significant reduction of thalamic perfusion during acute ischemic stroke of the anterior circulation. Without any evidence of ischemic damage to the thalamus on follow-up imaging, this finding is compatible with phenomena of the diaschisis complex. Parameters of thalamic hypoperfusion presented significant association with acute lesion extent on noncontrast CT and CTP, as well as ischemic involvement of the lentiform nucleus. Individual thalamic perfusion parameters displayed uncorrected significant association with clinical symptoms on admission but did not influence outcome at discharge or after 90 days.

The scaled parameters of non-ischemic thalamic hypoperfusion due to diaschisis showed significant perfusion reduction of the ipsilesional thalamus, compared to the contralateral side. Not all perfusion parameters were affected equally, with most prominent alterations of TTD and TMAX. Yet, the thalamic perfusion reduction did not reach levels of true ischemia as measured in the MCA territory. The slightly lower thalamic rCBV compared to the ischemic territory, is most likely explained by transient CBV increase of ischemic tissue outside the ischemic core (Murphy et al., 2006). Notably, we found a spectrum of thalamic hypoperfusion supporting our hypothesis that thalamic diaschisis does not necessarily represent a discrete entity that can be fully understood using a dichotomous classification.

This notion is further supported by our observations of significant linear interaction between thalamic hypoperfusion and acute lesion extent determined by CT perfusion and noncontrast CT. Accordingly, larger volumes of ischemia seem to induce a stronger reduction of ipsilateral thalamic perfusion. In an earlier study using visual classification of ITD, it has already been established, that ITD positive patients present larger ischemic volumes on CTP in the acute phase (Reidler et al., 2018). Here, we could provide first evidence of a direct interaction between lesion volume and grade of thalamic diaschisis in the acute phase of stroke.

Former studies in the acute and chronic phase of stroke have found a marked ischemic involvement of the basal ganglia in ITD positive patients (De Reuck et al., 1995, Ogawa et al., 1997, Reidler et al., 2018). In our multivariate regression model, only acute ischemic involvement of the lentiform nucleus, as part of the basal ganglia, presented independent association with more pronounced hypoperfusion expressed by longer ΔMTT, ΔTTD and ΔTMAX. The lentiform nucleus is comprised of the putamen and globus pallidus as part of the basal-ganglia loop, which also contains the thalamus as key hub. In this circuitry, the thalamus receives its main input from the globus pallidus internus, supporting our findings on a neuroanatomic level (Obeso et al., 2014, Goldberg et al., 2013). This further indicates that ischemic involvement of the basal ganglia and consequent disruption of the basal-ganglia loop are a key mechanism in the development of acute ITD.

Contrary to earlier findings that visual ITD status on CTP in the acute phase did not affect clinical presentation of stroke patients, we found a minor significant effect of rCBF on NIHSS on admission in uncorrected analysis, which did not maintain significance after correction for multiple comparisons. Therefore, this finding is of explorative nature. Further, no effect of thalamic perfusion was found on outcome at discharge and after 90 days. Other studies imply a negative clinical outcome in patients with positive ITD status in the subacute and chronic phase (Hendrik, 2020). This underlines the notion, that acute ITD does not necessarily reflect ITD in the chronic phase after stroke. The positive effect of endovascular thrombectomy and consequent recanalization as treatment might possibly dilute negative effects of acute ITD on long-term outcome in our data - especially compared to historical cohorts without timely reperfusion. Further investigation in larger numbers is therefore warranted to examine the course of ITD after stroke and its independent effect on clinical outcome measures. So far, no clear clinical implication for non-ischemic thalamic hypoperfusion or ITD has been established for the long-term outcome of patients receiving modern stroke treatment.

For thrombectomy triage, however, ITD represents a possible pitfall in the interpretation of CTP data as main guideline-based therapy selection criterion > 6 h after onset (Powers et al., 2019). DEFUSE 3 and DAWN study performed rigorous patient selection to include patients with small ischemic core and positive mismatch profile on software-based perfusion analysis with RAPID (iSchemaView). This third-party software is currently the only guideline-based analysis tool for perfusion imaging triage (Nogueira et al., 2018, Albers et al., 2018). Regarding these thresholds, we conclude, that on a global thalamic scale, misclassification of thalamic hypoperfusion due to diaschisis is unlikely for ischemic core and very rare for ischemic hypoperfusion (Mokin et al., 2017). Considering vendors using relative MTT to define ischemic hypoperfusion, caution is warranted as these thresholds were reached in 5/99 patients, which might influence estimation of the mismatch ratio. Comparing thalamic volume of around 5 mL (Craig et al., 2019) with median ischemic volume of 143 mL in our study, relevant impact on decision making in large-vessel occlusion stroke, however, seems unlikely. Notably, this analysis must be regarded as exploratory, as only raw cut-off values from different software packages were compared with our measurements.

This goes in hand with the limitation of this study that we cannot present analysis of non-ischemic thalamic hypoperfusion on a voxel or volumetric level, as we have performed our measurements in an axial region of interest. To our best knowledge, no software solution for thalamic segmentation on raw CTP data exists. Our comparisons with software thresholds are therefore of orienting character and need further, dedicated testing using the respective vendorś software. Still, we present the first standardized approach to quantify thalamic hypoperfusion during the acute phase of anterior circulation stroke on CTP.

Second, we focused on the examination of parameters of thalamic hypoperfusion on a continuous scale and did not differentiate variance of measurements from true diaschisis. Hence, CTP is only performed for symptomatic patients in emergency situations and includes considerable radiation exposure, no truly healthy controls can be provided as a study cohort. The exploratory analysis of patients with suspected stroke, but without ischemic changes on CTP indicates significantly lower thalamic perfusion in LVO stroke patients. However, the distribution of thalamic perfusion parameters in non-LVO patients needs further testing in larger cohorts,

Third, we can only provide incomplete data on short- and long-term clinical outcome. Yet our data on thalamic hypoperfusion represents the largest patient cohort so far, that received modern stroke treatment with endovascular thrombectomy, underlining the relevance of these findings in the present context of stroke management. Also, we included multiple clinical and imaging parameters to our multivariate regression model to identify a true independent association of thalamic hypoperfusion.

At last, we only provide a limited dataset with 99 patients. This is still among the largest cohorts for the study of diaschisis phenomena. Analysis in larger patient samples is needed to further refine the association between ITD and ischemic lesion or clinical parameters.

## 5 Summary/conclusion

Ipsilateral thalamic hypoperfusion is a non-binary phenomenon that occurs in acute ischemia of the anterior circulation and is affected by acute lesion extent and ischemic involvement of the lentiform nucleus. Additionally, parameters of hypoperfusion presented uncorrected association with clinical presentation on admission but not with short- or long-term outcome. Our data do not indicate risk of relevant misclassification as part of the ischemia proper using guideline-based thresholds in software analysis.

## Credit authorship contribution statement

**Paul Reidler:** Conceptualization, Methodology, Formal analysis, Investigation, Writing - original draft, Supervision. **Franziska Mueller:** Formal analysis, Investigation, Writing - original draft. **Lena Stueckelschweiger:** Formal analysis, Investigation, Writing - original draft. **Katharina Feil:** Conceptualization, Writing - review & editing. **Lars Kellert:** Conceptualization, Writing - review & editing. **Matthias P. Fabritius:** Formal analysis, Investigation, Writing - original draft. **Thomas Liebig:** Conceptualization, Methodology, Writing - original draft. **Steffen Tiedt:** Conceptualization, Writing - original draft. **Daniel Puhr-Westerheide:** Formal analysis, Investigation, Writing - original draft. **Wolfgang G. Kunz:** Conceptualization, Formal analysis, Investigation, Writing - original draft, Project administration.
