# Supplementary material for: Diaschisis revisited: quantitative evaluation of thalamic hypoperfusion in anterior circulation stroke
Source: Neuroimage Clin. 2020 Jun 26;27:102329. doi: 10.1016/j.nicl.2020.102329 (PMC7334597; doi:10.1016/j.nicl.2020.102329)
Supplement: Supplementary Data 1 [file mmc1.docx]

**ELECTRONIC SUPPLEMENTARY MATERIAL**

**Diaschisis Revisited: Quantitative Evaluation of Thalamic Hypoperfusion in Anterior Circulation Stroke**

**Supplemental Tables: 10**

**Supplemental Figures: 2**

**Supplemental Tables**

| **Supplemental Table I. Perfusion Parameters of Thalamus and Ischemic Territory in Patients Without Fetal PCA** | | | | | | | | | | | | | | | | | | | | | | | |
| --- | --- | --- | --- | --- | --- | --- | --- | --- | --- | --- | --- | --- | --- | --- | --- | --- | --- | --- | --- | --- | --- | --- | --- |
| **Absolute Measurements** |  | | | | | | |  | | | | | | | | | | | | | | |  |
| **n=90** | **Ipsilesional  Thalamus** | | | | | | | **Contralesional  Thalamus** | | | | | | | | | | | | | | | **p value** |
|  |  |  |  | | | | |  | | | |  | |  | | | | | | | | |  |
| CBF [mL/100g/min] | 54.0 |  | ( | 47.9 | – | 61.4 | ) | 71.1 |  | | ( | | | | 61.8 | | – | | 77.6 | | ) | | **<0.001*** |
| CBV [mL/100g] | 3.33 |  | ( | 3.06 | – | 3.68 | ) | 4.00 |  | | ( | | | | 3.56 | | – | | 4.34 | | ) | | **<0.001*** |
| MTT [s] | 3.93 |  | ( | 3.47 | – | 4.74 | ) | 3.49 |  | | ( | | | | 3.14 | | – | | 4.16 | | ) | | **0.002*** |
| TTD [s] | 3.44 |  | ( | 2.21 | – | 4.59 | ) | 2.42 |  | | ( | | | | 1.52 | | – | | 3.48 | | ) | | **<0.001*** |
| TMAX [s] | 1.52 |  | ( | 0.44 | – | 2.45 | ) | 0.74 |  | | ( | | | | 0.14 | | – | | 1.56 | | ) | | **<0.001*** |
|  |  |  |  |  |  |  |  |  |  | |  | | | |  | |  | |  | |  | |  |
| **n=90** | **Ischemic**  **Territory** | | | | | | | **Contralesional  Territory** | | | | | | | | | | | | | | | **p value** |
|  |  |  |  |  |  |  |  |  |  | |  | | | |  | |  | |  | |  | |  |
| CBF [mL/100g/min] | 27.79 |  | ( | 21.1 | – | 35.7 | ) | 59.64 |  | | ( | | | | 54.5 | | – | | 68.3 | | ) | | **<0.001*** |
| CBV [mL/100g] | 3.31 |  | ( | 2.79 | – | 3.71 | ) | 3.55 |  | | ( | | | | 3.31 | | – | | 3.78 | | ) | | **0.001*** |
| MTT [s] | 8.64 |  | ( | 7.18 | – | 10.5 | ) | 3.95 |  | | ( | | | | 3.43 | | – | | 4.60 | | ) | | **<0.001*** |
| TTD [s] | 11.98 |  | ( | 10.8 | – | 14.1 | ) | 3.44 |  | | ( | | | | 2.24 | | – | | 4.41 | | ) | | **<0.001*** |
| TMAX [s] | 7.89 |  | ( | 6.59 | – | 9.44 | ) | 1.41 |  | | ( | | | | 0.63 | | – | | 2.13 | | ) | | **<0.001*** |
|  |  |  |  | | | | |  | | | |  | |  | | | | | | | | |  |
| **Relative Measurements** |  |  |  | | | | |  | | | |  | |  | | | | | | | | |  |
| **n=90** | **Thalamus** | | | | | | | **Ischemic / Contralesional Territory** | | | | | | | | | | | | | | | **p value** |
|  |  |  |  | | | | |  | | | |  | |  | | | | | | | | | <0.001 |
| rCBF | 0.78 |  | ( | 0.70 | – | 0.89 | ) | 0.48 | |  | | | ( | | | 0.34 | | – | | 0.59 | | ) | **<0.001*** |
| rCBV | 0.84 |  | ( | 0.75 | – | 0.94 | ) | 0.91 | |  | | | ( | | | 0.78 | | – | | 1.03 | | ) | **0.012** |
| rMTT | 1.06 |  | ( | 1.00 | – | 1.17 | ) | 2.05 | |  | | | ( | | | 1.71 | | – | | 2.51 | | ) | **<0.001*** |
| rTTD | 1.29 |  | ( | 1.15 | – | 1.56 | ) | 3.43 | |  | | | ( | | | 2.73 | | – | | 5.30 | | ) | **<0.001*** |
| rTMAX | 1.66 |  | ( | 1.31 | – | 2.71 | ) | 5.40 | |  | | | ( | | | 3.88 | | – | | 11.0 | | ) | **<0.001*** |
| ∆MTT [s] | 0.22 |  | ( | 0.00 | – | 0.59 | ) | 4.40 | |  | | | ( | | | 3.11 | | – | | 5.66 | | ) | **<0.001*** |
| ∆TTD [s] | 0.70 |  | ( | 0.33 | – | 1.30 | ) | 8.89 | |  | | | ( | | | 7.43 | | – | | 10.4 | | ) | **<0.001*** |
| ∆TMAX [s] | 0.52 |  | ( | 0.23 | – | 0.93 | ) | 6.36 | |  | | | ( | | | 5.36 | | – | | 7.63 | | ) | **<0.001*** |
|  |  |  |  | | | | |  | | | |  | |  | | | | | | | | |  |
|  |  |  |  | | | | |  | | | |  | |  | | | | | | | | |  |
| Values presented are median (interquartile range). Nonparametric tests were performed using the Mann-Whitney U test Abbreviations: CBF, cerebral blood flow; CBV, cerebral blood volume; MTT, mean transit time; TTD, time to drain; rCBF / rCBV / rMTT / rTTD / rTMAX, relative ratio between ipsi- and contralesional measurements; ∆TTD / ∆MTT / ∆TMAX, absolute difference between ipsi- minus contralateral measurements. Bold numbers indicate p<0.05. * statistically significant after Bonferroni correction for 18 comparisons. | | | | | | | | | | | | | | | | | | | | | | | |

| **Supplemental Table II. Patient Characteristics of the Stroke Negative Group** | | | |
| --- | --- | --- | --- |
|  | **N=65** | | |
|  |  |  |  |
| **Patient Data** |  |  |  |
| Age | 69 |  | (57-78) |
| Female sex | 39 |  | (60.0%) |
| Time from symptom onset | 153 |  | (100-240) |
| NIHSS on admission | 2 |  | (0-3) |
| IV thrombolysis | 11 |  | (16.9%) |
|  |  |  |  |
| Consecutive patients who underwent multiparametric CT including CT perfusion but did not present ischemic changes on acute or follow-up imaging were selected. Values presented are count (percentage) for categorical and median (interquartile range) for ordinal or continuous variables. Time values are presented in minutes. Abbreviations: NIHSS, national Institute of Health Stroke Scale; IV, intravenous. | | | |

| **Supplemental Table III. Perfusion Parameters of the Thalamus in the Patient Group and Stroke Negative Group** | | | | | | | | | | | | | | | | | | | |  |
| --- | --- | --- | --- | --- | --- | --- | --- | --- | --- | --- | --- | --- | --- | --- | --- | --- | --- | --- | --- | --- |
|  | **LVO Stroke**  **Patients (N=99)** | | | | | | | | **Stroke Negative**  **Patients (N=65)** | | | | | | | | | | **p value** |  |
|  |  |  |  | | | | | |  | | |  |  | | | | | |  |  |
| rCBF | 0.78 |  | ( | 0.70 | – | 0.89 | | ) | 0.95 |  | ( | | | 0.92 | – | 0.98 | | ) | **<0.001*** |  |
| rCBV | 0.84 |  | ( | 0.75 | – | 0.92 | | ) | 0.95 |  | ( | | | 0.92 | – | 0.97 | | ) | **<0.001*** |  |
| rMTT | 1.06 |  | ( | 1.00 | – | 1.17 | | ) | 1.06 |  | ( | | | 1.02 | – | 1.10 | | ) | **0.94** |  |
| rTTD | 1.30 |  | ( | 1.16 | – | 1.56 | | ) | 1.08 |  | ( | | | 1.05 | – | 1.12 | | ) | **<0.001*** |  |
| rTMAX | 1.70 |  | ( | 1.38 | – | 2.70 | | ) | 1.13 |  | ( | | | 1.05 | – | 1.28 | | ) | **<0.001*** |  |
| ∆MTT [s] | 0.21 |  | ( | 0.01 | – | 0.55 | | ) | 0.22 |  | ( | | | 0.09 | – | 0.41 | | ) | **0.75** |  |
| ∆TTD [s] | 0.72 |  | ( | 0.34 | – | 1.25 | | ) | 0.26 |  | ( | | | 0.14 | – | 0.45 | | ) | **<0.001*** |  |
| ∆TMAX [s] | 0.53 |  | ( | 0.24 | – | 0.92 | | ) | 0.17 |  | ( | | | 0.05 | – | 0.29 | | ) | **<0.001*** |  |
|  |  |  |  |  |  |  | |  |  |  |  | | |  |  |  | |  |  |  |
| **Number of LVO Stroke Patients with Perfusion Parameters ≥ or ≤ Stroke Negative Average±2SD** | | | | | | | | | | | | | | | | | | | |  |
| **Cut-off Parameter** | **Raw # (%)** | | | | | | | | | | | | | | | | | | | |
|  |  |  |  | | | | | | | | | | | | | | | | | |
| rCBF ≤ Average-2SD | 62 | | | | | | ( | | | | | | 63.3% | | | | ) | | | |
| rCBV ≤ Average-2SD | 61 | | | | | | ( | | | | | | 61.2% | | | | ) | | | |
| rMTT ≥ Average+2SD | 23 | | | | | | ( | | | | | | 23.2% | | | | ) | | | |
| rTTD ≥ Average+2SD | 57 | | | | | | ( | | | | | | 57.6% | | | | ) | | | |
| rTMAX ≥ Average+2SD | 35 | | | | | | ( | | | | | | 35.4% | | | | ) | | | |
| ∆MTT [s] ≥ Average+2SD | 20 | | | | | | ( | | | | | | 20.2% | | | | ) | | | |
| ∆TTD [s] ≥ Average+2SD | 45 | | | | | | ( | | | | | | 45.5% | | | | ) | | | |
| ∆TMAX [s] ≥ Average+2SD | 38 | | | | | | ( | | | | | | 38.4% | | | | ) | | | |
|  |  |  |  | | | | | |  | | |  |  | | | | | |  |  |
| Values presented are median (interquartile range). Nonparametric tests were performed using the Mann-Whitney U test. Relative values in the stroke negative group were calculated using the hemispheric measurements with smaller CBF or CBV and longer MTT, TTD, and TMAX as numerator. Abbreviations: LVO, large vessel occlusion; CBF, cerebral blood flow; CBV, cerebral blood volume; MTT, mean transit time; TTD, time to drain; rCBF / rCBV / rMTT / rTTD / rTMAX, relative ratio between ipsi- and contralesional measurements; ∆TTD / ∆MTT / ∆TMAX, absolute difference between ipsi- minus contralateral measurements; SD, standard deviation. Bold numbers indicate p<0.05. * statistically significant after Bonferroni correction for 8 comparisons. | | | | | | | | | | | | | | | | | | | |  |

| **Supplemental Table IV. Software based CT perfusion-thresholds** | | | | |
| --- | --- | --- | --- | --- |
|  | **Ischemia** | | **Ischemic Core** | |
|  |  |  |  |  |
| **N=99** | **Cut-off**  **value** | **Reached in thalamic ROI measurements** | **Cut-off**  **value** | **Reached in thalamic ROI measurements** |
|  |  |  |  |  |
| RAPID  (iSchemaView) | TMAX >6s | 1 (1%) | rCBF <0.3 | 0 (0%) |
| Syngo Via Neuro Perfusion  (Siemens healthineers, Forchheim, Germany) | CBF <35.1mL/100g/min | 1 (1%) | CBV <1.2mL/100g | 1 (1%) |
| Brain CT Perfusion Package  (Philips Healthcare, Best, The Netherlands) | rMTT >1.45 | 5 (5%) | rMTT >1.45 and CBV  <2.0 mL/100g | 0 (0%) |
| CT perfusion thresholds for ischemia and infarction core of the indicated software packages. Cases with thalamic perfusion measurements in our positive for the indicated parameters are presented as raw numbers and percentage in parenthesis. Abbreviations: rCBF, relative cerebral blood flow; rMTT, relative mean transit time; CBV, cerebral blood volume. | | | | |

| **Supplemental Table V. Association of Thalamic Perfusion with Acute Imaging Parameters** | | | | | | | | | | |
| --- | --- | --- | --- | --- | --- | --- | --- | --- | --- | --- |
| **N=99** | **Thalamic  rCBF** | | **Thalamic  rCBV** | | **Thalamic  ∆MTT** | | **Thalamic  ∆TTD** | | **Thalamic  ∆TMAX** | |
|  |  |  |  |  |  | |  | |  | |
| **Independent variables** | **β** | **p value** | **β** | **p value** | **β** | **p value** | **β** | **p value** | **β** | **p value** |
|  |  |  |  |  |  |  |  |  |  |  |
| Total ischemic volume | -0.15 | 0.20 | -0.05 | 0.66 | 0.25 | **0.04** | 0.34 | **0.004^†^** | 0.36 | **0.002^†‡^** |
| Ischemic core volume | -0.08 | 0.55 | -0.08 | 0.52 | -0.02 | 0.90 | 0.06 | 0.66 | 0.05 | 0.69 |
| Noncontrast CT ASPECTS | 0.12 | 0.30 | 0.27 | **0.02** | 0.17 | 0.15 | 0.10 | 0.36 | 0.09 | 0.45 |
| Fetal PCA | -0.06 | 0.58 | -0.10 | 0.33 | -0.04 | 0.68 | -0.03 | 0.80 | -0.02 | 0.86 |
|  |  |  |  |  |  | |  | |  | |
| A multivariate linear regression analyses were performed for the indicated acute imaging parameters. Abbreviations: CBF, cerebral blood flow; CBV, cerebral blood volume; MTT, mean transit time; TTD, time to drain; rCBF / rCBV, relative CBF / CBV as ratio between ipsi- and contralesional measurements; ∆TTD / ∆MTT / ∆TMAX, absolute difference between ipsi- minus contralateral measurements, ASPECTS, Alberta Stroke Program Early CT Score, PCA, posterior cerebral artery. Bold numbers indicate p<0.05. † statistically significant after Bonferroni correction across 5 regression models. ‡ statistically significant after Bonferroni correction for 20 parameters. | | | | | | | | | | |

| **Supplemental Table VI. Association of Thalamic Perfusion with Acute Imaging Parameters in Patients Without Fetal PCA** | | | | | | | | | | |
| --- | --- | --- | --- | --- | --- | --- | --- | --- | --- | --- |
| **n=90** | **Thalamic  rCBF** | | **Thalamic  rCBV** | | **Thalamic  ∆MTT** | | **Thalamic  ∆TTD** | | **Thalamic  ∆TMAX** | |
|  |  |  |  |  |  | |  | |  | |
| **Independent Variables** | **β** | **p value** | **β** | **p value** | **β** | **p value** | **β** | **p value** | **β** | **p value** |
|  |  |  |  |  |  |  |  |  |  |  |
| Total ischemic volume | -0.24 | **0.02** | -0.18 | 0.09 | 0.19 | 0.07 | 0.34 | **0.001^†‡^** | 0.36 | **<0.001^†‡^** |
| Ischemic core volume | -0.23 | **0.03** | -0.26 | **0.01** | 0.03 | 0.79 | 0.19 | 0.08 | 0.20 | 0.06 |
| Mismatch % | 0.17 | 0.11 | 0.24 | **0.03** | 0.06 | 0.61 | -0.06 | 0.58 | -0.70 | 0.51 |
| Noncontrast CT ASPECTS | 0.23 | **0.03** | 0.36 | **0.001^†‡^** | 0.11 | 0.29 | -0.24 | 0.82 | -0.46 | 0.67 |
|  |  |  |  |  |  | |  | |  | |
| Univariate linear regression analyses were performed for the indicated acute imaging parameters. Abbreviations: CBF, cerebral blood flow; CBV, cerebral blood volume; MTT, mean transit time; TTD, time to drain; rCBF / rCBV, relative CBF / CBV as ratio between ipsi- and contralesional measurements; ∆TTD / ∆MTT / ∆TMAX, absolute difference between ipsi- minus contralateral measurements, ASPECTS, Alberta Stroke Program Early CT Score, PCA, posterior cerebral artery. Bold numbers indicate p<0.05. † statistically significant after Bonferroni correction across 5 regression models. ‡ statistically significant after Bonferroni correction for 20 parameters. | | | | | | | | | | |

| **Supplemental Table VII. Stroke Topography by Affected ASPECTS Regions** | | | | |
| --- | --- | --- | --- | --- |
| **N=99** | **Regional CT-Perfusion-Based Acute Ischemia** | | **Regional Final Infarction** | |
|  |  |  |  |  |
| **Region** | **Raw #** | **(%)** | **Raw #** | **(%)** |
|  |  |  |  |  |
| Caudate Nucleus | 46 | (46.5%) | 48 | (48.5%) |
| Internal Capsule | 45 | (45.5%) | 33 | (33.3%) |
| Insula | 91 | (91.9%) | 58 | (58.6%) |
| Lentiform Nucleus | 48 | (48.5%) | 60 | (60.6%) |
| M1 Cortex | 85 | (85.9%) | 27 | (27.3%) |
| M2 Cortex | 95 | (96.0%) | 34 | (34.3%) |
| M3 Cortex | 79 | (79.8%) | 25 | (25.3%) |
| M4 Cortex | 78 | (78.8%) | 38 | (38.4%) |
| M5 Cortex | 95 | (96.0%) | 38 | (38.4%) |
| M6 Cortex | 83 | (83.3%) | 20 | (20.2%) |
|  |  |  |  |  |
| Data are presented as raw # (percentage). Regional ischemia was determined on CT perfusion imaging, Final Infarction was determined on follow-up. Regional ischemia was rated as present if ≥20% of its territory was affected. Abbreviations: ASPECTS, Alberta Stroke Early CT Score; M1 – M6 Cortex, cortical regions of the ASPECTS. | | | | |

| **Supplemental Table VIII. Clinical Parameter Analysis** | | | | | | |
| --- | --- | --- | --- | --- | --- | --- |
|  | **Admission**  **NIHSS (N=99)** | | **Discharge**  **mRS (n=83)** | | **90-Day**  **mRS (n=70)** | |
|  |  |  |  |  |  |  |
| **Independent**  **Variables** | **OR**  **(95%-CI)** | **p value** | **OR**  **(95%-CI)** | **p value** | **OR**  **(95%-CI)** | **p value** |
|  |  |  |  |  |  |  |
| Noncontrast CT | 0.70  (0.52 – 0.93) | **0.01^†^** |  |  |  |  |
| Ischemic core volume | 0.98  (0.97 – 1.00) | **0.01^†^** |  |  |  |  |
| Age | 0.99  (0.97 – 1.02) | 0.64 | 1.04  (1.02 – 1.07) | **0.003^†‡^** | 1.06  (1.02 – 1.10) | **0.001^†‡^** |
| Sex | 0.98  (0.42 – 2.32) | 0.97 | 0.92  (0.40 –2.13) | 0.82 | 0.69  (0.27 – 1.78) | 0.60 |
| Total ischemic volume | 1.02  (1.01 – 1.03) | **<0.001^†‡^** | 1.01  (1.00–1.01) | **0.02** | 1.00  (1.00 – 1.01) | 0.53 |
| Final Infarction Volume |  |  | 1.01  (1.01 – 1.02) | **0.001^†‡^** | 1.02  (1.01 – 1.03) | **0.02^†^** |
| mTICI |  |  | 6.40  (2.33 – 17.3) | **<0.001^†‡^** | 2.87  (1.01 – 8.15) | **0.048** |
| Intravenous  Thrombolysis |  |  | 1.45  (0.57 – 3.66) | 0.44 | 0.99  (0.38 – 2.62) | 0.99 |
|  |  |  |  |  |  |  |
| A multivariate ordinal logistic regression analysis was performed for the indicated parameters. Abbreviations: NIHSS, National Institutes of Health Stroke Scale; mRS, modified Rankin Scale; CBF, cerebral blood flow; CBV, cerebral blood volume; MTT, mean transit time; TTD, time to drain; rCBF / rCBV, relative CBF / CBV as ratio between ipsi- and contralesional measurements; ∆TTD / ∆MTT / ∆TMAX, absolute difference between ipsi- minus contralateral measurements; OR, odds ratio; mTICI, modified Treatment in Cerebral Ischemia score. Bold numbers indicate p<0.05. † statistically significant after Bonferroni correction across 3 regression models. ‡ statistically significant after Bonferroni correction for 17 parameters. | | | | | | |

| **Supplemental Table IX. Association of Thalamic Perfusion with Clinical Parameters for Patients with TICI 2b/3** | | | | |
| --- | --- | --- | --- | --- |
|  | **Discharge**  **mRS (n=59)** | | **90-Day**  **mRS (n=49)** | |
|  |  |  |  |  |
| **Independent**  **Variables** | **OR**  **(95%-CI)** | **p value** | **OR**  **(95%-CI)** | **p value** |
|  |  |  |  |  |
| rCBF | 0.08  (0.01 – 5.73) | 0.25 | 0.09  (0.01 – 99.9) | 0.98 |
| rCBV | 0.26  (0.01 – 18.0) | 0.53 | 1.22  (0.01 – 122) | 0.93 |
| ∆MTT [s] | 0.99  (0.50 – 1.97) | 0.97 | 0.81  (0.37 – 1.78) | 0.59 |
| ∆TTD [s] | 0.82  (0.51– 1.30) | 0.39 | 0.81  (0.48 – 1.38) | 0.44 |
| ∆TMAX [s] | 0.73  (0.39 – 1.34) | 0.31 | 0.79  (0.49 – 1.56) | 0.50 |
|  | | | | |
| A multivariate ordinal logistic regression analysis was performed for the indicated parameters. Further variables for Discharge and 90-day mRS included Age, Sex, Total ischemic volume, final infarction volume, intravenous therapy. Abbreviations: mRS, modified Rankin Scale; CBF, cerebral blood flow; CBV, cerebral blood volume; MTT, mean transit time; TTD, time to drain; rCBF / rCBV, relative CBF / CBV as ratio between ipsi- and contralesional measurements; ∆TTD / ∆MTT / ∆TMAX, absolute difference between ipsi- minus contralateral measurements; OR, odds ratio; mTICI, modified Treatment in Cerebral Ischemia score. Bold numbers indicate p<0.05. | | | | |

| **Supplemental Table X. Association of Thalamic Perfusion with Clinical Parameters for Patients with TICI 0-2a** | | | | |
| --- | --- | --- | --- | --- |
|  | **Discharge**  **mRS (n=24)** | | **90-Day**  **mRS (n=21)** | |
|  |  |  |  |  |
| **Independent**  **Variables** | **OR**  **(95%-CI)** | **p value** | **OR**  **(95%-CI)** | **p value** |
|  |  |  |  |  |
| rCBF | 0.17  (0.01 – 98.8) | 0.58 | 0.08  (0.01 – 56.3) | 0.35 |
| rCBV | 0.48  (0.01 – 303) | 0.82 | 0.04  (0.01 – 40.3) | 0.36 |
| ∆MTT [s] | 3.28  (0.90 – 11.8) | 0.07 | 3.56  (0.63 – 20.1) | 0.15 |
| ∆TTD [s] | 3.12  (1.00– 9.74) | 0.051 | 2.92  (0.55 – 15.5) | 0.21 |
| ∆TMAX [s] | 3.05  (0.86 – 10.9) | 0.09 | 3.38  (0.38 – 29.9) | 0.28 |
|  | | | | |
| A multivariate ordinal logistic regression analysis was performed for the indicated parameters. Further variables for Discharge and 90-day mRS included Age, Sex, Total ischemic volume, final infarction volume, intravenous therapy. Abbreviations: mRS, modified Rankin Scale; CBF, cerebral blood flow; CBV, cerebral blood volume; MTT, mean transit time; TTD, time to drain; rCBF / rCBV, relative CBF / CBV as ratio between ipsi- and contralesional measurements; ∆TTD / ∆MTT / ∆TMAX, absolute difference between ipsi- minus contralateral measurements; OR, odds ratio; mTICI, modified Treatment in Cerebral Ischemia score. Bold numbers indicate p<0.05. | | | | |

**Supplemental Figures and Figure Legends**

**Supplemental Figure I.** Flow chart of patient selection. Abbreviations: CTP, CT perfusion

**Supplemental Figure II.** Scatterplots of thalamic perfusion parameters in relation to overall acute ischemic volume on CTP. Displayed are individual values (blue), trendline (black) and 95%-confidence interval (red).
